# A case report of bilateral synovial chondromatosis of the ankle

**DOI:** 10.1186/1746-1340-15-18

**Published:** 2007-11-24

**Authors:** Heather Shearer, Paula Stern, Andrew Brubacher, Tania Pringle

**Affiliations:** 1 Department of Graduate Education and Research, Canadian Memorial Chiropractic College, Toronto, Canada; 2 Private practice, Brooklin, Canada; 3 Department of Radiology, Canadian Memorial Chiropractic College, Toronto, Canada

## Abstract

**Background:**

Synovial chondromatosis is a rare, generally benign condition which affects synovial membranes. It most commonly involves large joints such as the knee, hip, and elbow, but its presence in smaller joints has also been reported. The diagnosis of synovial chondromatosis is commonly made following a thorough history, physical examination, and radiographic examination. Patients may report pain and swelling within a joint which is often aggravated with physical activity.

**Case presentation:**

A rare case of bilateral synovial chondromatosis of the ankle is reviewed. A 26 year-old male presented with chronic bilateral ankle pain. Physical examination suggested and imaging confirmed multiple synovial chondromatoses bilaterally, likely secondary to previous trauma.

**Conclusion:**

The clinical and imaging findings, along with potential differential diagnoses, are described. Since this condition tends to be progressive but self-limiting, indications for surgery depend on the level of symptomatic presentation in addition to the functional demands of the patient. Following a surgical consultation, it was decided that it was not appropriate to pursue surgery at the present time.

## Background

Synovial chondromatosis is an uncommon disorder of unknown aetiology and is characterized by the presence of multiple cartilaginous nodules in the joint synovium or cavity [1,2]. Although often benign, malignant transformation can occur [3]. It typically presents unilaterally in large joints such as the knee but can occur in the shoulder, elbow, hip, ankle and temporomandibular joints [4,5]. Synovial chondromatosis is more common in males, and current literature cites symptomatic presentation predominantly ranging from the third to fifth decade [1,6,7]. The diagnosis of synovial chondromatosis is given after a thorough history, physical examination, and radiographic examination. However, the definitive diagnosis is achieved after histological examination of the synovial tissue [4]. The treatment of choice for symptomatic patients is surgical [2,4]. There is debate in the literature regarding arthroscopic versus open-procedures and whether the synovium should be removed [2,4]. Conservative management of symptomatic individuals has not been reported in the literature. We describe an unusual presentation of bilateral synovial chondromatosis in the ankle joint.

## Case presentation

### Clinical history

A 26 year-old male student presented to a chiropractic clinic with a complaint of chronic bilateral ankle pain. Walking was not limited by pain, although he reported sharp constant pain that was located over the dorsum of the ankles. The pain intensity varied from 2/10 to 7/10. The pain was aggravated by physical activity such as running and relieved by rest and ice. There was a previous history of locking and swelling in both ankles. The locking was usually accompanied by decreased active dorsiflexion until the patient manually self-mobilized the ankle to regain the lost motion. He reported recurrent bilateral ankle sprains over the past few years. The patient was otherwise healthy and past medical history and systems review were unremarkable.

### Physical examination

On examination, the patient weight was within normal limits with respect to his height. No lower limb alignment abnormalities or leg length inequalities were noted. There was no swelling or redness. He had difficulty heel walking due to left ankle pain. Right ankle active and passive ranges of motion were decreased by 10% in dorsi- and plantar flexion. Resisted testing was unremarkable. Neurological examination of the lower limb was unremarkable. Orthopaedic examination illustrated positive bilateral anterior drawer and synovial impingement manoeuvres. The impingement manoeuvre involved concurrent pressure applied anterior and inferior to the lateral maleolus while the ankle was moved from plantar to dorsiflexion [8]. One leg stance was held for 5 and 10 seconds on the right and left, respectively. Joint play illustrated restrictions in the right subtalar joint. Muscle palpation revealed tight bilateral soleus, fibularis and anterior tibialis muscles with no noted asymmetry in muscle mass.

The patient was diagnosed with bilateral synovial hypertrophy with associated ligamentous laxity. He received conservative treatment which included ultrasound, soft tissue therapy, ankle joint manipulation and exercises. After eight visits over the course of one month, no improvement was noted and plain film radiographs of the right ankle were ordered.

**Figure 1:**
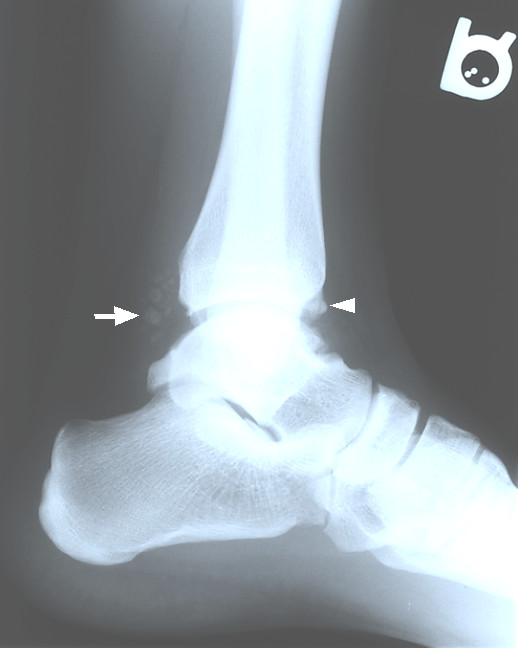
Lateral right ankle radiograph with evidence of calcified loose bodies (arrow) posterior to the talotibial joint. Small loose bodies are also seen anteriorly to the joint (arrow head).

The radiographs illustrated several calcific loose bodies projecting posterior to the tibiotalar joint with additional loose bodies anterior to the joint. The ankle mortise and subtalar joint spaces were well maintained. A small osteophyte was noted at the posterior malleolus (Figure 1). Mild degenerative joint disease of the tibiotalar joint was also noted.

The diagnosis was changed to primary synovial chondromatosis potentially associated with a history of previous trauma to the joint. Conservative care was discontinued at this point.

Five months later the patient returned with acute left ankle pain following an inversion injury six days earlier while playing indoor soccer. At the time of injury there was immediate pain and swelling. The patient was able to weight bear within 10 minutes. He reported limping for the first few days following the injury.

On examination, swelling was evident at the left lateral malleolus. Active and passive left ankle ranges of motion were painful and decreased by 25%. The talar tilt test on the left was positive. Anterior drawer was positive on the right with no pain. This test was difficult to perform on the left due to swelling and pain. Palpation elicited tenderness anterior to the lateral maleolus and the fibularis longus muscle. One legged stance was not painful for greater than 10 seconds.

**Figure 2:**
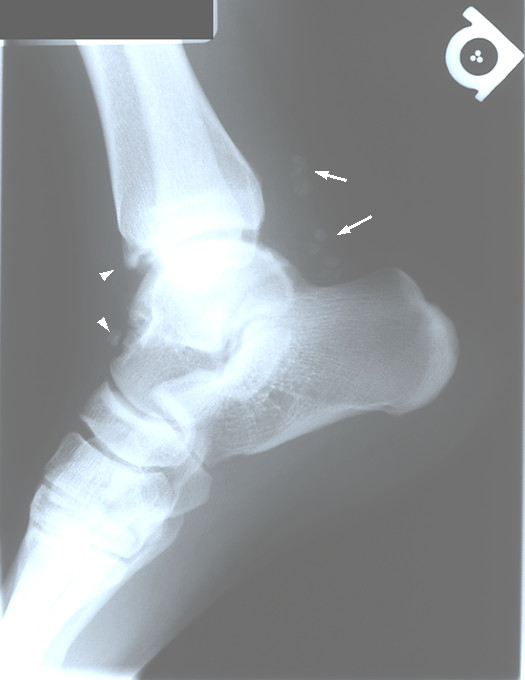
Left lateral ankle view demonstrating multiple calcified loose bodies likely located in both the flexor hallucis and tibialis posterior tendons (arrow). Loose bodies are also present anterior to the talotibial joint (arrow head).

**Figure 3:**
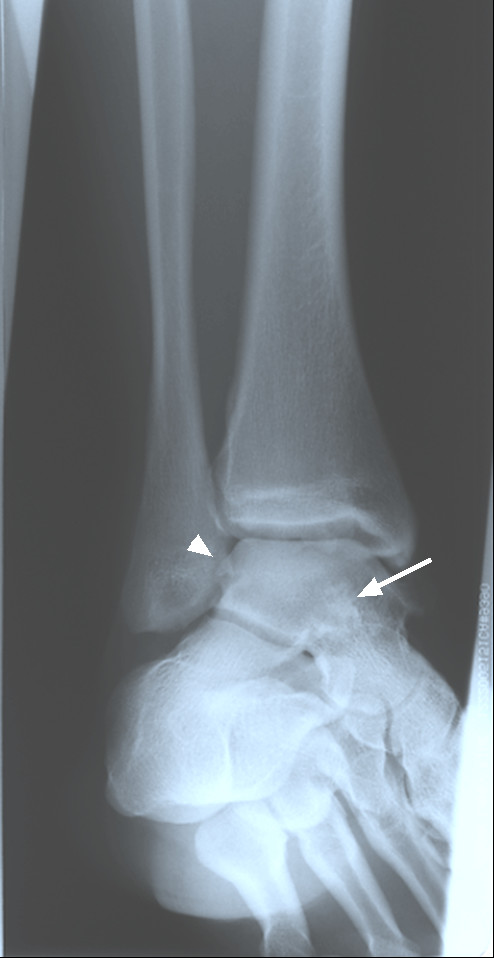
Oblique left ankle radiograph with evidence of calcified loose bodies medial (arrow head) to the lateral maleolus and superimposed over the talus (arrow). This suggests synovial chondromatosis, likely located in both the flexor hallucis and tibialis posterior tendons.

Radiographs of the left ankle were ordered. These illustrated bone-spurring at the medial and anterior talofibular joint (Figures 2 &3). Several ossified bodies with lucent centres were noted posterior and anterior to the talotibial joint. It was suggested that the calcific bodies were located in the synovial sheath of the flexor hallucis longus or tibialis posterior tendon. Soft-tissue swelling was detected anterior and posterior to the talotibial joint. Mild degenerative joint changes at the anterior and medial talotibial joint, likely traumatic in origin, were noted (Figures 2 &3). As with the right ankle, the patient was diagnosed with primary synovial chondromatosis, likely associated with a history of previous joint trauma.

### Management

The patient began treatment which included cryotherapy, ultrasound, and soft tissue work to the fibularis muscles. Manual mobilizations of the ankle mortise joint occurred infrequently. He received six treatments over approximately 4 weeks. The patient was discharged as asymptomatic with minor residual swelling and some periodic episodes of locking.

**Figure 4:**
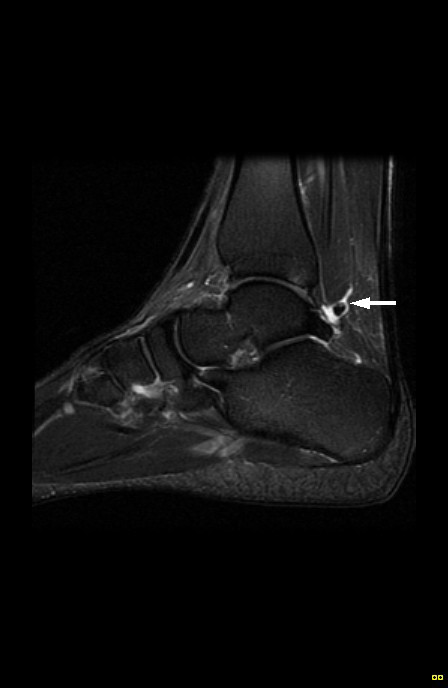
Sagittal MRI of the right ankle (fat-saturated T2-weighted) revealing a predominantly low signal intensity nodule in the synovial sheath of the flexor hallucis longus tendon (arrow).

**Figure 5:**
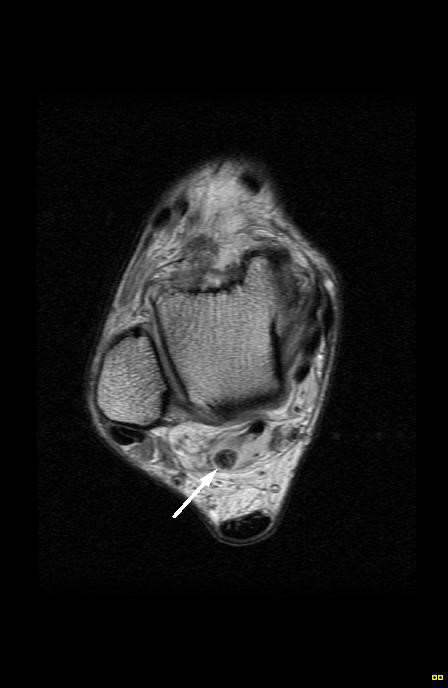
Axial MRI of the right ankle (proton density) revealing a (arrow) heterogeneous nodule of low and intermediate signal intensities located in the flexor hallucis longus tendon sheath. Of interest is the degree of distension of the tendon sheath secondary to the surrounding effusion.

**Figure 6:**
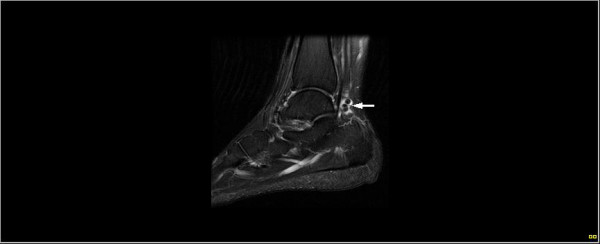
Sagittal MRI of the left ankle (fat-saturated T2-weighted) illustrates (arrow) two distinct low signal intensity nodules with surrounding effusion posterior to the talo-tibial joint.

**Figure 7:**
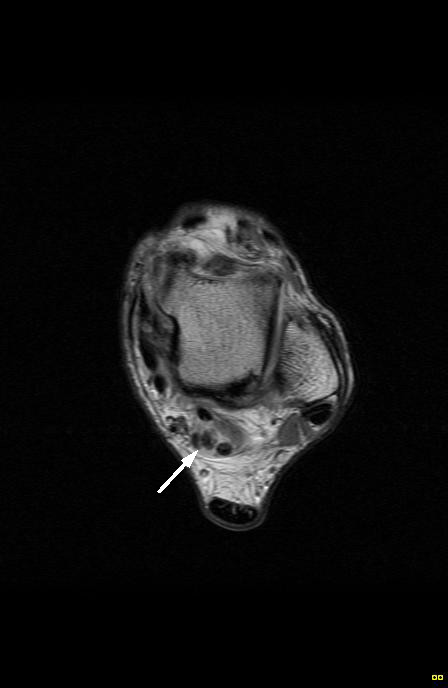
Axial MRI of the left ankle (proton density) demonstrates (arrow) multiple heterogeneous and low signal intensity nodules within the extended flexor hallucis longus tendon sheath.

Although discharged from conservative care, the patient was referred for an orthopaedic surgery consult due to the recurrent nature of the ankle pain and the radiographic findings. MR imaging was ordered and revealed calcifications in both ankle joints and the right and left flexor hallucis longus tendon sheaths (Figures 4, 5, 6, 7). Following MR imaging and orthopaedic assessment, the orthopaedic surgeon concluded that surgery to extract the calcifications from the tendons would be too invasive and would not be pursued at the present time. The patient was advised to continue with his daily activities.

## Discussion

Synovial chondromatosis is a rare benign condition characterized by the presence of cartilaginous nodules in the synovium of joints, tendon sheaths, and bursae which often occur without trauma or inflammation [1,9,10]. With disease progression, the loose bodies may ossify and can be identified radiographically [11]. There are a variety of names for this lesion. The most commonly accepted include synovial chondromatosis, synoviochondrometaplasia, synovial chondrosis, synovial osteochondromatosis, and articular chondrosis [2,11].

The condition is generally thought to be monoarticular and over 50% of reported cases occur in the knee [6,12]. Other locations include the hip, elbow, shoulder, and ankle joints, although any synovial joints can be affected [7,13,14]. Synovial chondromatosis is usually identified in the third to fifth decades of life and is rarely seen in children [6,7]. It is more commonly identified in males, with almost a two-to-one ratio in comparison with women [2,11]. The onset is described as insidious and occurs over months to years [2]. Iossifidis et al described an insidious, non-specific clinical presentation in their case of ankle synovial chondromatosis [6].

It is generally agreed that the exact aetiology of synovial chondromatosis is unknown and controversy exists surrounding proposed hypotheses. Milgram, in 1977, categorized the disease process into 3 distinct phases [15]. In phase I, metaplasia of the synovial intima occurs. Active synovitis and nodule formation is present, but no calcifications can be identified. In phase II, nodular synovitis and loose bodies are present in the joint. The loose bodies are primarily still cartilaginous. In phase III, the loose bodies remain but the synovitis has resolved. The loose bodies also have a tendency to unite and calcify [15]. Because there is no evidence of histologic metaplasia in stage three, diagnosis may be more difficult.

Despite the varied nomenclature, it is recognized that synovial chondromatosis can be differentiated into a primary and secondary form. The primary form occurs in an otherwise normal joint [4]. Primary synovial chondromatosis is characterized by undifferentiated stem cell proliferation in the stratum synoviale [16]. The pathological process is considered to be a cartilaginous metaplasia of synovial cells with trauma commonly thought of as an inciting stimulus, although no statistical relationship has been reported in the literature. Via immunostaining, it has been concluded that primary synovial chondromatosis is a metaplastic condition [17]. The individual nodules may detach from the synovium and form loose bodies in the joint. These loose bodies may continue to grow, being nourished by the synovial fluid. These nodules can continue on to calcify, known as osteochondromatosis, although it is reported that calcification is only present in 2/3 of patients. Some have hypothesized that this form is actually a secondary disorder following cartilage shedding into a joint [18]. Primary synovial chondromatosis is generally thought to be progressive, more likely to recur, and may lead to severe degenerative arthritis with long-term presence [11,12].

Secondary synovial chondromatosis is thought to be caused by irritation of the synovial tissue of the affected joint [4,14]. It occurs when cartilage fragments detach from articular surfaces and become embedded in the synovium. These loose bodies are nourished by the synovium, induce a metaplastic change in the subsynovium, and consequently produce chondroid nodules [14]. This form is associated with degenerative joint disease, trauma, inflammatory and non-inflammatory arthropathies, avascular necrosis, and osteochondritis dissecans [14]. This form is not likely to recur following surgical removal [11].

Recent interest in this diagnosis has occurred due to the potential for malignant degeneration. Although rare, there are a number of reported cases and patients diagnosed with this condition should be monitored [3]. In a 1998 study examining primary synovial chondromatosis, a relative risk of 5% for malignant degeneration was reported [19]. The progression of synovial chondromatosis to chondrosarcoma is very rare and some may argue it is simply a case of misdiagnosis. Nonetheless, a distinction between these two entities may be difficult. Clinical and radiographic features of these conditions are similar. As such, clinical, radiographic or advanced imaging, and histological evidence should be considered collectively to arrive at an accurate diagnosis.

The diagnosis of synovial chondromatosis is often made following a thorough history, physical examination, and radiographic examination. Patients may report pain and swelling within a joint. This is routinely exacerbated with physical activity. Commonly, the patient may also report aching, reduced range of motion, palpable nodules, locking, or clicking of the joint [7,11]. These lesions may become symptomatic following mechanical compression or irritation of soft tissues, nerves, or malignant transformation. In rare cases, reactive bursas can form over osteochondromas. These may be another source of pain, but can also mimic chondrosarcoma [14]. Conversely, individuals may have no signs or symptoms and it is an incidental finding secondary to another complaint. According to Milgram, this is related to the stage of the lesion [15].

According to Milgram's classification, plain film radiographs are only helpful in the third phase of the disease, once calcification has occurred [15]. Advanced imaging, such as CT and MRI scans are useful in identifying and localizing the lesions as well as helping to distinguish between other differential diagnoses. When imaging does not provide specific diagnostic features, it is important to obtain a tissue biopsy. A definitive diagnosis is made histologically via a synovial tissue biopsy. Blood tests and arthritis profiles can also help rule out specific differential diagnoses.

Potential differential diagnoses include osteochondritis dissecans, synovial vascular malformation, pigmented villonodular synovitis, chondrosarcoma, injury-related soft-tissue calcification, and lipoma arborescence with osseous metaplasia [4,20].

Since the condition tends to be progressive but self-limiting, indications for surgery depend on the level of symptomatic presentation in addition to the functional demands of the patient [6]. In asymptomatic patients, the nodules may resorb over time and invasive procedures should be avoided [4]. Patient age and disease stage may also serve as treatment guides. In young patients, arthroscopic debridement is commonly sufficient to achieve a cure and synovectomies should be used only in instances of relapse [10]. In phase III disease, removal of the loose bodies alone is sufficient [13]. Resection of the loose bodies and synovectomy when synovitis is present is thought to be indicated since the recurrence is increased when synovitis is present [13]. Recurrence rates for synovial chondromatosis after surgical treatment have been reported as varying from 7% to 23% [2]. Overall, prognosis following removal of the nodules is reported as excellent. Although no comparative studies for the ankle have been performed, removal of loose bodies and synovectomy of the knee produced good results in function, pain, and control of synovitis in 90% of subjects [21].

If the diagnosis is not definitive, it is recommended to biopsy and debride initially. Surgery predisposes patients to tissue scarring, subsequently compromising joint function. If disabling symptoms are persistent, arthrodesis is a reasonable approach [1,6].

## Conclusion

This case is reported because of its rarity. No other cases of bilateral ankle synovial chondromatosis have been reported, especially involving calcific nodules in tendon sheaths bilaterally. Lack of awareness of this condition may lead to incorrect diagnoses and unwarranted surgery. Because of the concern of chondrosarcoma, if radiographic or MR imaging are inconclusive, a histological diagnosis is a prudent course for this condition.

## Abbreviations

CT: computerized tomography

DJD: degenerative joint disease

MR: magnetic resonance

## Competing interests

The author(s) declare that they have no competing interests.

## Authors' contributions

HMS performed a literature search and helped draft and revised the manuscript. PS participated in the coordination of the report and helped draft and revise the manuscript. AB participated in the collection of information and helped draft the manuscript. TP reviewed all imaging and revised the manuscript. All authors read and approved the final manuscript.
